# VCAb: a web-tool for structure-guided exploration of antibodies

**DOI:** 10.1093/bioadv/vbae137

**Published:** 2024-09-20

**Authors:** Dongjun Guo, Joseph Chi-Fung Ng, Deborah K Dunn-Walters, Franca Fraternali

**Affiliations:** Institute of Structural and Molecular Biology, University College London, London WC1E 6BT, United Kingdom; Randall Centre for Cell and Molecular Biophysics, School of Basic and Medical Biosciences, King’s College London, London SE1 1UL, United Kingdom; Institute of Structural and Molecular Biology, University College London, London WC1E 6BT, United Kingdom; School of Biosciences and Medicine, University of Surrey, Guildford GU2 7XH, United Kingdom; Institute of Structural and Molecular Biology, University College London, London WC1E 6BT, United Kingdom; Department of Biological Sciences, Birkbeck, University of London, London WC1E 7HX, United Kingdom

## Abstract

**Motivation:**

Effective responses against immune challenges require antibodies of different isotypes performing specific effector functions. Structural information on these isotypes is essential to engineer antibodies with desired physico-chemical features of their antigen-binding properties, and optimal developability as potential therapeutics. *In silico* mutational scanning profiles on antibody structures would further pinpoint candidate mutations for enhancing antibody stability and function. Current antibody structure databases lack consistent annotations of isotypes and structural coverage of 3D antibody structures, as well as computed deep mutation profiles.

**Results:**

The *V* and *C* region bearing *a*nti*b*ody (VCAb) web-tool is established to clarify these annotations and provides an accessible resource to facilitate antibody engineering and design. VCAb currently provides data on 7,166 experimentally determined antibody structures including both V and C regions from different species. Additionally, VCAb provides annotations of species and isotypes with numbering schemes applied. These information can be interactively queried or downloaded in batch.

**Availability and implementation:**

VCAb is implemented as a R shiny application to enable interactive data interrogation. The online application is freely accessible https://fraternalilab.cs.ucl.ac.uk/VCAb/. The source code to generate the database and the online application is available open-source at https://github.com/Fraternalilab/VCAb.

## 1 Introduction

Antibodies, a key component of the immune system, are composed of two pairs of heavy (H) chain and light (L) chain, with each chain bearing variable (V) and constant (C) regions (Dreyer and Bennett 1965, Lu *et al.* 2018, Chiu *et al.* 2019, Guo *et al.* 2024). The V region engages with the antigen through the complementary-determining regions (CDRs), which exhibit high variability due to somatic hypermutation, enhancing the affinity to antigens. In contrast, the C region is relatively constant and designates the H or L chain identity: light chain can be either κ or λ while there are nine heavy chain types (Guo *et al.* 2024). The types of heavy chain define the “isotype” of the antibody and determine its relevance in different immune processes: IgM and IgD are predominant in a primary response, IgG are the most prevalent antibodies in plasma, IgE is seen in allergic reactions, and IgA is responsible for the immune response in mucosal membranes. Isotypes of antibodies can be switched by a process called class-switch recombination, where only the C region of the heavy chain is changed but remains untouched (Janeway *et al.* 2001).

The first therapeutic antibody, muromonab-CD3, was approved by the US FDA in 1986 to treat acute transplant rejection (Lu *et al.* 2020). Since then, antibodies are broadly applied as therapeutics because of their specificity to the targeted antigens and the effector functions they trigger to coordinate immune clearance of such antigens (Zinn *et al.* 2023). For example, cetuximab is a therapeutic antibody used for treating head-and-neck and bowel cancers (Concu and Cordeiro 2018). It functions by binding to the epidermal growth factor receptor (EGFR) and preventing cancer cell migration and invasion. Rituximab targets CD20 on malignant B cells in lymphoma treatment and uses its Fc region to trigger antibody-dependent cellular cytotoxicity (ADCC; Salles *et al.* 2017). The main focus of research in the field of antibody engineering has been to evolve the binding affinity to the antigen by changing the V region, due to its direct role in the engagement of the antigen (Tabasinezhad *et al.* 2019, Hie *et al.* 2024). Unstable antibodies showed impeded or lost efficacy, high chances of aggregation, low production yield, and low propensity to become developable therapeutic antibodies (Ma *et al.* 2020, Hanning *et al.* 2022). To this end, biophysical, energy-based approaches and machine-learning-based methods have been used to generate large-scale *in silico* predictions of mutations to improve antibody thermostability (Leaver-Fay *et al.* 2011, Ruffolo *et al.* 2021, Cheng *et al.* 2023, Harmalkar *et al.* 2023). However, studies have highlighted the importance of the C region in modulating antigen interactions (Cooper *et al.* 1993, Torres *et al.* 2007, Casadevall and Janda 2012, Tudor *et al.* 2012, Lua *et al.* 2018, Khamassi *et al.* 2020, Guo *et al.* 2024) and fulfilling antibody stability and function, underscoring the necessity to consider entire antibody structures in antibody engineering strategies. In addition, the development of next-generation sequencing methods has allowed for a deep sampling of the antibody repertoire of many individuals, profiling a great diversity of V regions coupled with different isotypes (Marks and Deane 2020, Olsen *et al.* 2022). This raises questions on the properties of these antibodies at the protein structural level, and the scope to engineer both V and C regions to improve the binding functions and stability of the antibody. Current antibody-specific structural modeling applications allow for fast and accurate structural modeling of the V regions (Ruffolo *et al.* 2023, Kenlay *et al.* 2024). However, structural prediction of the antibody assembly, including both V and C regions is missing, but this is important for dissecting the antigen-binding and effector functions of antibodies, as discussed earlier. To answer these questions, collection and annotation of antibody structures containing both V and C regions, with different types of H chain and L chain, are needed.

**Figure 1.**
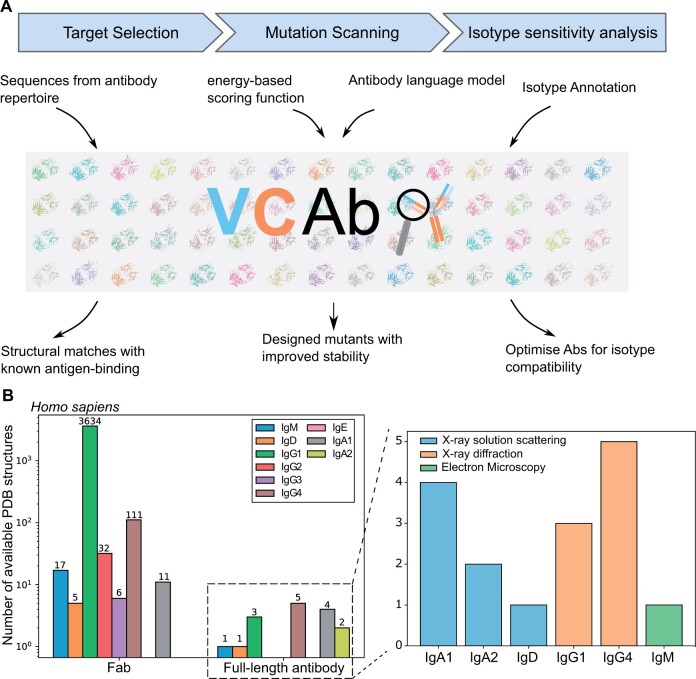
VCAb: a user-friendly web tool for structure-guided exploration of antibodies. (**A**) VCAb offers functionalities to facilitate the exploration of antibody structures, including target selection, mutational scanning, and isotype sensitivity analysis. (**B**) Experimental human antibody structures of different isotypes, structural coverages are collected in VCAb. The bar charts show (*left*) statistics on the coverage of human antibody structures and (*right*) the isotype distribution of full-length human antibody structures, as of 19 August 2024.

A number of databases are available for interrogating antibody structural data, such as Protein Data Bank (PDB; Berman *et al.* 2003), IMGT (ImMunoGeneTics information system)/3Dstructure-DB (Kaas *et al.* 2004, Ehrenmann and Lefranc 2011) and SAbDab (Dunbar *et al.* 2014, Schneider *et al.* 2022), amongst others. While the PDB encompasses the breadth of protein structures, it lacks an easy-to-use query interface for the user to specifically filter antibody structures. On the other hand, other well-established databases like IMGT/3Dstructure-DB do not offer a programmatic interface to access data in bulk, limiting queries to one structure at a time, precluding large-scale analyses pipeline integration. A recently developed antibody-based queryable database, SAbDab (Dunbar *et al.* 2014, Schneider *et al.* 2022), was specifically built to offer accurate annotation of the V regions of antibody structures. To the best of our knowledge, currently available antibody databases do not provide information of the effect on mutations. To address the gap for reliable, large-scale, and easily retrievable annotations of both V and C region structures and their *in silico* mutational scanning profiles, we present VCAb (*V* and *C* region-bearing *a*nti*b*ody database). VCAb collates experimentally resolved antibody structures with both V and C regions; the database (i) is readily updated and easily queried, (ii) contains clear information about the sequence, isotype/light chain type, and structural coverage, (iii) offers, in addition to V region sequences conforming to the IMGT numbering scheme provided by other standard tools (Abhinandan and Martin 2008, Giudicelli *et al.* 2011, Dunbar and Deane 2016), IMGT-gapped C region sequences, allowing consistent analysis of structural features such as domain packing geometries, and uniquely, (iv) *in silico* mutational scanning data for experimental antibody structures which can be of useful help in designing stable antibodies. VCAb can be queried online (https://fraternalilab.cs.ucl.ac.uk/VCAb/) using characteristics such as isotype, sequence similarity, or CH1–CL interface similarity, with the mutational scanning profiles displayed for the user-selected entry (Supplementary Materials S1). Other databases [such as SAbDab (Dunbar *et al.* 2014), etc.] also offer the functionality of searching antibody structures by sequence similarity. However, to our knowledge, no other antibody-specific databases provide all these features in a one-stop-shop style and allow for programmatic access to the data, making it the first fully open-source antibody structural database. VCAb can serve as an ideal tool for researchers interested in the selection of template for antibody modeling purposes, and the structural properties of the antibody to optimize the geometries and stabilities of antibody designs (Fig. 1A), as these characteristics are crucial for antigen binding (Fernández-Quintero *et al.* 2020) and the effectiveness of the antibody (Ma *et al.* 2020).

## 2 Implementation

### 2.1 Data collection

SEQRES (https://www.wwpdb.org/documentation/file-format) records of protein chains with resolved structures were automatically downloaded from worldwide PDB archive (Burley *et al.* 2019). This is important as it represents the true structural coverage of the antibody (Supplementary Fig. S1). Antibody sequences with both V and C regions were identified from the downloaded protein sequences using ANARCIvc, a package we modified from ANARCI (Dunbar and Deane 2016) to number sequences of both V and C regions conforming to IMGT rules (Supplementary Materials S3.1.3). IMGT rules are developed to assign a unique number to antibody sequence, in order to highlight structurally or functionally important positions and allowing for consistent comparison across multiple antibodies (Lefranc *et al.* 2003, 2005). Sequences successfully analyzed and numbered using ANARCIvc were deemed to contain the correct sequence features expected for antibody V and C regions. The downloaded mmCIF antibody structure files were annotated with structural metadata using the PDBe (Armstrong *et al.* 2020) application programming interface (API); all procedures were automated in a set of python scripts available in the VCAb github repository. As of 19 August 2024, 7166 antibodies had been collected in VCAb, 3676 of which are human (downloaded on 19 August 2024, Fig. 1B). This data collection process has been automated to update the database monthly.

### 2.2 Features annotation

VCAb annotates antibody species, isotype, and structural coverage by comparing its sequence to all IMGT (Lefranc *et al.* 2015) reference alleles using BLAST (Altschul *et al.* 1990). To address spurious species annotation provided by the PDBe API while mitigating potential artifacts arising from BLAST local alignments, we considered each V/C domain separately, consolidated both annotations and overwrote the PDBe-provided species if the best BLAST hit has a percentage of identity larger by 8% compared to the PDBe annotation; we observed that this cutoff effectively separated antibody V and C sequences from different species (Supplementary Materials S3.1.2). We further consolidated the nominated species for each domain to generate a final annotation that flagged different engineered formats (humanized, chimera). Isotype and light chain type were identified to be the BLAST hit with highest percentage identity. To ensure the accurate assignment of structural coverage, the sequence representing residues with ATOM (https://www.wwpdb.org/documentation/file-format) records extracted from mmCIF was used as input to BLAST alignments against IMGT reference alleles. We classified structures as Fab or full antibody, depending on whether coordinates for CH2, CH3, or CH4 were included in the structure. We provided the following annotations of Fab structures: (i) packing angles (elbow angle, CH1–CL angle) as defined by Fernández-Quintero *et al.* (2020); (ii) annotations of interface between heavy and light chains (“H–L interface”) derived using POPSCOMP (Kleinjung and Fraternali 2005); and (iii) contact matrix considering the Cα-Cα distances between all residues along the heavy and light chains. All CH1 and CL sequences were numbered using ANARCIvc (Supplementary Materials S3.1.3) so that all downstream analyses conform to the IMGT standard numbering scheme for the C region for ease of comparison.

### 2.3 In silico antibody mutational scanning

We applied three *in silico* mutational scanning methods to evaluate the impact of mutations on antibodies: this includes (i) Rosetta point mutant scan application (Leaver-Fay *et al.* 2011) yielding structure-based scores focusing on the physico-chemical features of residues; (ii) pseudo-log-likelihood from the AntiBERTy (Ruffolo *et al.* 2021), a language model for the antibody V region of antibody. We scaled the raw AntiBERTy score of every point mutation by subtracting the AntiBERTy score for the wild type (WT) residue at each position, such that they can be interpreted as relative changes in amino acid preference compared to WT (Supplementary Materials S3.2.2). This method has been applied to predict mutational effects on protein function (Meier *et al.* 2021). (iii) AlphaMissense (Cheng *et al.* 2023) scores to evaluate mutational effects of C-region mutations. These methods were applied on any possible amino acid substitution in every VCAb entry to constitute *in silico* mutational scanning datasets, with all of them freely accessible for download.

### 2.4 VCAb web server

The VCAb website has been built to allow data access for academic research purposes, available at https://fraternalilab.cs.ucl.ac.uk/VCAb/. The website displays the features described above for each VCAb entry, enables filtering of antibody structures based on these features, and supports searches by sequence similarity, using BLAST (Altschul *et al.* 1990) accessed via the rBLAST package (version 0.99.2). This search is flexible to the region of interest (V region or both V and C regions) and both paired and unpaired H/L chain sequences, and supports input of single sequences, uploads of multiple FASTA sequences (maximum 200 per batch), as well as tabulated antibody repertoire data in standard formats (AIRR (Adaptive Immune Receptor Repertoire) standard tab-separated files, comma-separated files, and output from single-cell repertoire sequencing analysis generated by the 10x Genomics Cellranger software; Supplementary Materials S1.3). Users can also search VCAb by CH1–CL interface similarity, which is derived by comparing the contact matrix at the CH1–CL interface generated for each VCAb entry (Supplementary Materials S1.2.2).

The VCAb webserver provides visualization functionalities for 3D structures, structural coverage, antibody numbering information for both V and C regions, as well as tabulated details of H–L interface, disulfide bonds, and *in silico* mutational scanning results. The interactive 3D viewer enables detailed inspection of the structure. The following metadata are displayed for each VCAb entry: PDB and chain identifiers, assigned germline CH and CL alleles, structural coverage, and species. A list of detailed information included in VCAb can be found in the online documentation accessible in the “About” page on the online interface; these additional columns can be accessed either by customizing the table view to show these additional columns, or by bulk downloads of search results and/or the entire VCAb database in comma separated value (CSV) format.

## 3 Application

### 3.1 Investigation of COVID-19 repertoire to illustrate the binding between antibody and RBD domain

The volume of sequences obtained via high-throughput sequencing of the antibody repertoire is rapidly expanding, yielding paired heavy and light chain immunoglobulins attributed to single B cells (Olsen *et al.* 2022) collected from vaccinations and disease scenarios (Schultheiß *et al.* 2020, Jin *et al.* 2021, Kotagiri *et al.* 2022, Stewart *et al.* 2022). Each single-cell sequencing experiment can sample approximately $10^{5}-10^{6}$ antibodies, posing a significant challenge to obtain all structures at the repertoire level both experimentally and computationally. Even for the cutting-edge antibody modeling pipeline ImmunoBuilder (Abanades *et al.* 2023), which operates at an impressive speed of 5 seconds per structure, it would still require a week to 58 days to complete all computations. Furthermore, only a small portion of antibodies can interact with a specific antigen, making the structural modeling of the entire antibody repertoire unnecessary. VCAb, as a structural annotation web server, can be usefully exploited to identify the best structural homologs for chosen repertoire sequences, by searching via sequence similarity through the experimentally determined structures present in the database. This prescreening of the repertoire prioritizes antibodies for further analysis, such as structural modeling, docking, or antibody–antigen modeling software, potentially aiding in the interpretation of their functional properties (e.g. antibody–antigen interaction).

**Figure 2.**
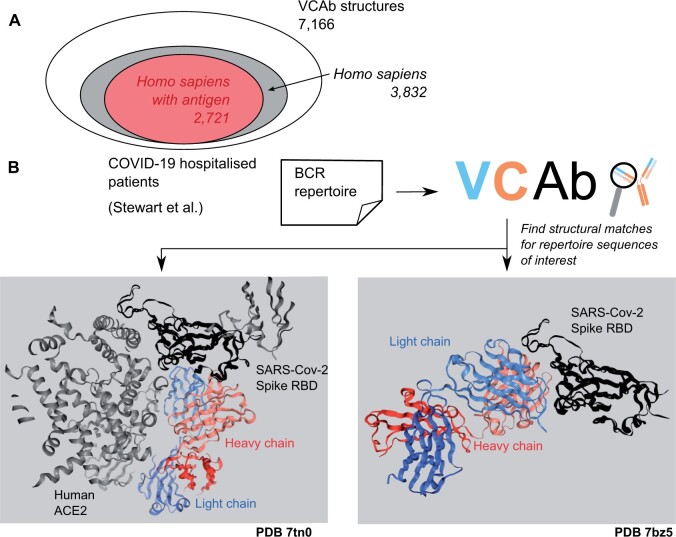
Investigation of COVID-19 repertoire from a structural perspective. (**A**) Antibody structural space in VCAb. The majority (53.5%) of antibody structures in VCAb is from human, among which 2721/3832 (71.0%) of these are co-complexed with antigens. (**B**) Two sequences from the sampled repertoire (Stewart *et al.* 2022) are inputted into VCAb to search for matches in the dataset of resolved antibody structures. We obtained a structural match (*left*, PDB 7tn0) showing the recognition of the RBD epitope distinct from ACE2 binding site. For the other sequence (*right*), the corresponding structure match (PDB 7bz5) shows this antibody directly blocking ACE2 binding. RBD, receptor binding domain.

As an example, we asked whether the availability of antigen-antibody complex structures in VCAb can be harnessed to annotate antibody sequences sampled during an immune response (Fig. 2A). We used repertoire data from Stewart *et al.* (2022) of hospitalized coronavirus disease 2019 (COVID-19) patients, and applied VCAb to search for structural matches and illustrate how the sampled antibody sequences bind to the antigen, the spike protein S1 of severe acute respiratory syndrome coronavirus 2 (SARS-CoV-2). The top four structural hits for the sampled sequence show high sequence similarity (95.8%) for the V region, with all of them containing the antigen (spike protein S1), which indicates the likely antigen for this selected antibody sequence from the repertoire (Supplementary Fig. S3B). Another sampled sequence gives rise to multiple structural hits with sequence identity above 94%. All of them contain the S1 protein, suggesting the identity of the antigen for the selected sequence entry (Supplementary Fig. S3C). As a reference, the sequence similarity between two antibodies with identical sequences except for the CDR region results in 72.7% sequence identity. A stricter comparison between antibodies of the same sequence except CDR3, a region crucial for antibody–antigen binding, yields 89.8% sequence identity. The experimental structures mapped exceeded both criteria.

When we used the structural viewer functionality provided in VCAb to compare the top-ranked structure matches (7bz5_HL and 7tn0_MN) for these two selected repertoire sequences, we observed that they interact with the same antigen differently. 7bz5_HL binds to the cellular receptor ACE2 (Angiotensin-converting enzyme 2) binding site of the spike receptor binding domain (RBD), making contact with most residues on the RBD-ACE2 interface for its competition with ACE2 (Wu *et al.* 2020). On the other hand, 7tn0_MN binds to the cryptic epitope on the other side of the RBD domain without occupying the ACE2 binding site (McCallum *et al.* 2022, Fig. 2B). Here, VCAb functions as a prescanning tool for repertoire sequences, highlighting potential sequences of the user’s interest. This allows the user to apply more sophisticated tools in subsequent analysis for more accurate predictions and interpretation of antigen-binding properties in the sampled repertoire sequences, thereby facilitating the engineering of antibodies to efficiently engage the antigen (Piccoli *et al.* 2020, Hummer *et al.* 2022).

### 3.2 *In silico* mutational scanning of antibody structures

*In vitro* deep mutational scanning to measure the impact of point mutations is a costly and time-consuming pipeline involving protein display, screening and sequencing, etc. (Hanning *et al.* 2022). Here, VCAb offers *in silico* predictions of the effect on single point mutations for each antibody structure generated using different methods: an energy-based method relying on the antibody structure (Rosetta; Leaver-Fay *et al.* 2011), and machine-learning-based methods [using the antibody-specific language model AntiBERTy (Ruffolo *et al.* 2021) for V region and the AlphaMissense model (Cheng *et al.* 2023) for C region]. Rosetta calculates a score that has been demonstrated to correlate well with changes in the Gibbs free energy of unfolding (ΔG) due to mutations, indicating changes in protein stability (Sora *et al.* 2023). Thermostability has significant implications for the design of therapeutic antibodies (Hanning *et al.* 2022). Therefore, we utilize changes in experimentally determined melting temperature (ΔTm) as an indicator to assess the effectiveness of Rosetta in predicting the consequences of mutations. The antibody-specific language model encapsulates sequence variation constraints by evaluating the likelihood of each amino acid occurring at every position, based on observed examples during training (Chungyoun *et al.* 2024). These sequence variation constraints arise from various factors, including thermostability, antigen-binding, self-tolerance, etc., providing the user a richer prospective on the impact of mutations. These mutational scanning profiles in VCAb can aid in prioritizing promising mutations for subsequent experimental characterization.

**Figure 3.**
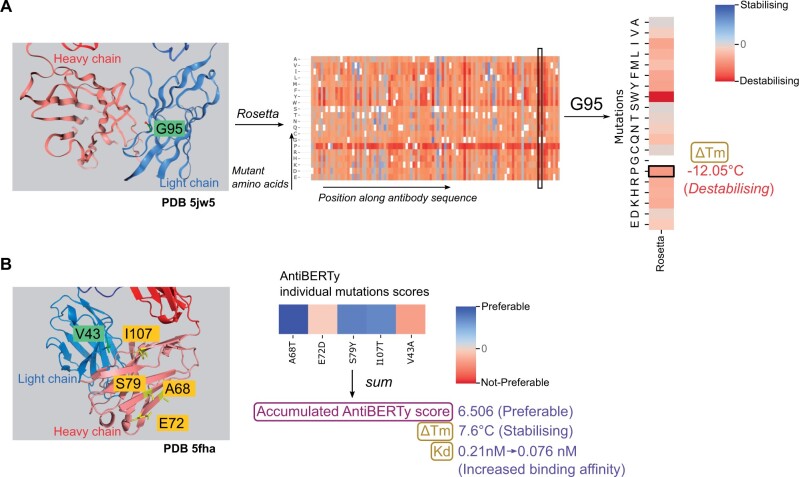
*In silico* mutational scanning of antibody structures. (**A**) Rosetta scores for antibody (PDB 5jw5) at residue G95. Experimental measurement of the ΔTm for G95P shows a negative value (i.e. this mutation is destabilizing to the antibody). The position G95 is highlighted (ball-and-stick in green) on the 3D structure is shown on the left. (**B**) An example of antibody structure (PDB 5fha) with multiple mutations highlighted. Mutations on both heavy and light chains are labeled. Pseudo-log-likelihood for individual mutations are added together to estimate the effect of introducing multiple mutations together. Experimental measurements of this quintuple mutant show the changes of melting temperature of 7.6 °C, indicating its stabilizing effect; and decreasing of the $K_{d}$ to the antigen ebolavirus glycoprotein, indicating the increase in antigen binding affinity. ΔTm, change in melting temperature; $K_{d}$, dissociation constant.

MEDI8852 is an antibody-neutralizing influenza A hemagglutinin (Hie *et al.* 2024) with an experimentally resolved structure (PDB 5jw5). Figure 3A shows the scores calculated from Rosetta, with red being destabilizing and blue being stabilizing. To validate the utility of these data, we compared these scores against a recent analysis (Hie *et al.* 2024) which used a protein language model (ESM-1b) to design novel mutations on its unmutated common ancestor (UCA) with high sequence similarity, and validated their impact experimentally. The mutation G95P in the VL domain was found to be destabilizing (decrease in melting temperature [Tm]) but its affinity to the antigen was enhanced (Hie *et al.* 2024). Analyzing this in VCAb, this mutation is predicted to be destabilizing using Rosetta, which is consistent with its experimental validation with its negative ΔTm. In fact, most mutations at this position are predicted as unpreferable, with a few predicted as neutral by Rosetta. Glycine has the smallest side chain among all the aminoacids, and by inspecting it in the 3D structural viewer, it sits at the interface between VH and VL domain. This indicates the importance of the spatial localization of this residue at the VH–VL interface and can act as the starting point for further analysis.

For each residue in the V region of any VCAb structure, both Rosetta and AntiBERTY scores are provided to users for comparing the effects of using different types of information (sequence and structure) in predicting mutational impact. We note that for Rosetta, since each point mutation was predicted separately, the resultant scores are not additive and therefore would not be suitable for predicting the effect of multiple mutations in combination. Instead, AntiBERTy, being a language-model-based method, can address this issue: since each amino acid is represented as a “word” in a sentence, the pseudo-log-likelihood returned by the model can be summed together to represent the likelihood of observing several amino acids in combination (Supplementary Fig. S9), in the same way as the likelihood of a given sentence being presented is evaluated in language models used in natural language processing (NLP; Shin *et al.* 2019, Salazar *et al.* 2020, Meier *et al.* 2021). This would capture the overall impact of the multiple mutations to antibody functions, with the assumption that each mutation occurs independently. mAb114 is an antibody binding to the glycoprotein of ebolavirus (PDB ID 5fha). A quintuple mutant (heavy chain: A68T, E72D, S79Y, I113T; Light chain: V43A) has been designed with improved affinity to the antigen (Hie *et al.* 2024). Three out of the five single mutations are predicted as preferable by AntiBERty (Fig. 3B). Summing over the pseudo-log-likelihood of individual mutations yields a positive accumulated score for the co-occurrence of the five mutations together, indicating that it is preferable for the five mutations being presented at the same time, when compared with the wild type. Experimental measurements validate this prediction, showing a positive ΔTm in thermostability and a nearly three-fold reduction in $K_{d}$ value, a critical parameter demonstrating the enhanced binding affinity and functionality of the antibody (Fig. 3B).

### 3.3 Exploring the consequence of isotype switching on antibody structural stability

**Figure 4.**
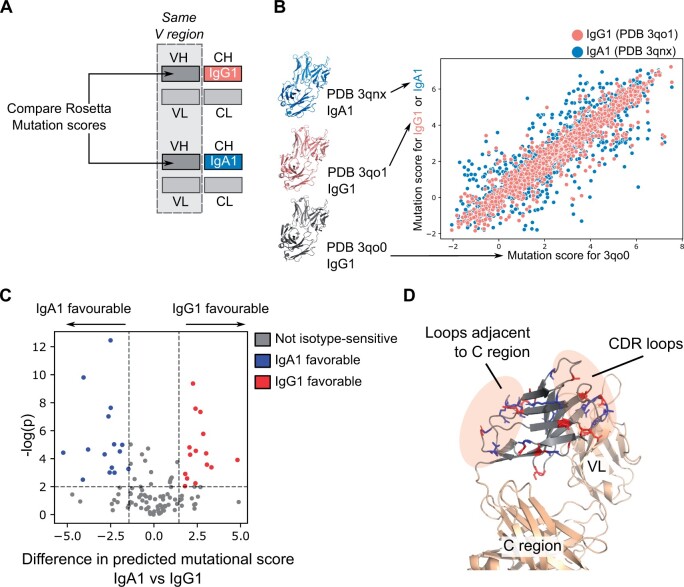
Exploring the consequence of isotype switching on antibody structural stability. (**A**) Rosetta mutational scanning scores for VH residues from antibodies of different isotypes are compared. These antibodies have the same VH and light chain sequences, the only difference between them is their CH1 domain. (**B**) Scatter plot of Rosetta mutational scanning scores. Dots correspond to the correlation between Rosetta mutational scores of structures of the same isotype (IgG1: PDB ID 3qo0 and PDB ID 3qo1) and structures of different isotypes (IgG1: PDB ID 3qo0 and IgA1: PDB ID 3qnx) are displayed. (**C**) A volcano plot is derived for each position in the VH domain by comparing the difference in the mutation scores calculated from the IgA1 versus the IgG1 structure (see Supplementary Materials S2.3). (**D**) The IgG1/IgA1-sensitive positions highlighted in panel (**C**) are visualized on the antibody structure. Most of them concentrate on CDR loops and loops close to C region.

Therapeutic antibody design requires careful selection of isotypes to achieve desired downstream effector functions (Zinn *et al.* 2023). This is relevant also for *in vivo* antibody maturation, where isotype switching is a critical process to adapt the antibody to function in different contexts (Janeway *et al.* 2001). However, how isotype switching would affect antibody stability was poorly investigated. Benefiting from the mutational scanning analysis and isotype annotation of the antibody structures in VCAb, users can begin investigating the hot spots in the V region, which are sensitive (in terms of the changes of stability) upon coupling with different C regions, and potentially engineer these hot spots to stabilize the antibody. Here we analyzed isotype switching *in silico*, by making use of a set of Fab structures in VCAb with identical V regions (originally isolated from a lymphoma patient; Houdayer *et al.* 1993) coupled with both IgA1 (PDB 3qnx) and IgG1 (PDB 3qo0; Fig. 4A). Comparing the Rosetta pmut scan results of the VH region, the method agrees on mutational impact at most positions in the two structures, although Rosetta scores are able to discriminate between isotypes at specific positions (Fig. 4B). Using another IgG1 structure with the same V region (PDB ID 3qo1) as the negative control (Fig. 4B), we considered the mutational impact upon switching isotypes to detect locations sensitive to the selection of isotypes (Fig. 4C, see Supplementary Materials S2.3). The fold difference between the IgA1-IgG1 comparison over the IgG1-IgG1 comparison is calculated to indicate the favorable isotype, with negative values meaning mutations at this position tend to be IgA1-favorable and positive values being IgG1-favorable. We hypothesize that these positions represent either functionally important residues, or regions in proximity to the C region. In Fig. 4D, we display VH residues with mutations showing significant differences in the scores calculated in the context of different isotypes. Most of these positions are located in the loops near the C region, highlighting how the proximity of the V and C regions together determines structural stability. Interestingly, some residues on the CDRH3 loop are also highlighted from this analysis. Previous research discovered that antibodies with the same V region but different isotypes have distinct affinity to the antigen (Casadevall and Janda 2012, Tudor *et al.* 2012). This analysis helps prioritize positions for further investigation into the relationship between antigen binding affinity and the sensitivity of these positions to isotype switching.

## 4 Conclusion

VCAb harmonizes annotations of antibody isotypes, species, and structural coverage, and provides data for detailed analysis to improve antibody design. The VCAb database is updated once a month to include newly released, experimentally determined antibody structures. Users can interact with the online webserver for accessing annotations of individual structures, or for batch downloads of these annotations over many structures; alternatively, users can also use the publicly available source code to build and maintain a local version of the database for offline usage.

Engineering antibodies involves optimizing various characteristics of therapeutic antibodies, such as thermostability, antigen-binding, or self-tolerance. We show that *in silico* mutational scanning profiles produced by different methods in VCAb can aid antibody engineering, as confirmed by various experimental measurements collected from publications. However, we have noticed that more advanced tools, such as language models, are now available to accurately predict the effects of mutations on different properties, such as thermostability. Fine-tuning the model for thermostability prediction requires extensive experimental data on antibodies (e.g. melting temperature), which is currently unavailable due to commercial interests and is time-consuming. A recent paper exhibits state-of-the-art performance in predicting antibody thermostability by integrating structural information from Rosetta with language models (Hutchinson *et al.* 2024). While the trained weights of this model and the experimental data used for training are not available, this paper highlights the effectiveness of combining language models (which encompass sequence variation constraints) with biophysical methods (specifically Rosetta) for particular user-defined tasks. We incorporate both types of information in VCAb, allowing users to conduct further analysis or combine scores from both methods to create custom tools for different antibody engineering tasks. To evaluate the impact of multiple mutations, AntiBERTy might serve as a solution by combining the pseudo-log-likelihoods at each specific position, assuming that each mutation occurs independently. However, this assumption of independence does not always hold, especially for compensatory mutations, thus limiting AntiBERTy’s capacity to forecast the effects of multiple mutations.

A number of precomputed characteristics of antibodies in VCAb enable users to delve into the structural properties of antibodies. For instance, through the clarification of isotype annotations, users can further explore the relationship between antibody V and C regions. The *in silico* mutational scanning profiles will inform users on how to optimize antibody design. We foresee that VCAb, as the first fully open-sourced antibody structural database, will provide informative guidelines for future tailored investigations of antibody design and engineering, as well as the selection of homology modeling templates to investigate the structures of different antibody isotypes.

## Supplementary Material

vbae137_Supplementary_Data

## Data Availability

VCAb is freely accessible at https://fraternalilab.cs.ucl.ac.uk/VCAb/. The source code to generate the VCAb database and the online R shiny application is available at https://github.com/Fraternalilab/VCAb. The package ANARCIvc developed on the top of ANARCI is available at https://github.com/Fraternalilab/ANARCI_vc.
